# Thinking Outside the Box—and the Thoracic Cage: The Rise of Subcostal Single‐Port Robotic Lung Surgery

**DOI:** 10.1002/wjs.70412

**Published:** 2026-05-08

**Authors:** Shah‐Hwa Chou, Yu‐Wei Liu

**Affiliations:** ^1^ Division of Thoracic Surgery Department of Surgery Kaohsiung Medical University Hospital Kaohsiung Medical University Kaohsiung Taiwan

The evolution of minimally invasive thoracic surgery has been a relentless pursuit of “less is more”. From open thoracotomy to multi‐port video‐assisted thoracoscopic surgery (VATS) and subsequently to robot‐assisted thoracic surgery (RATS), each step has sought to reduce surgical trauma while maintaining oncological rigor.

Despite the widespread adoption of multi‐port RATS (such as the da Vinci Xi), several unmet needs persist in the field of thoracic oncology. Although RATS offers superior visualization and dexterity, the requirement for multiple intercostal incisions—typically four to five—still poses a risk for intercostal nerve injury and persistent postsurgical pain. Conventional uniportal VATS addressed this by using a single incision, but at the expense of the 3D visualization and wrist‐like articulation that robotic surgeons have come to rely on.

In 2022, Diego Gonzalez‐Rivas' research established “Pure Uniportal RATS” as a major advancement, integrating robotic precision with single 3–4 cm incisions for complex thoracic procedures without rib spreading. Key breakthroughs included adapting multi‐arm platforms for single‐port access and executing total console control for dissection and stapling [1].

Recently, the advent of the da Vinci SP (Single‐Port) system has pushed the boundaries of this philosophy, promising a true uniportal experience. In pilot studies of SP lung resection, Lee and Cheng et al. respectively demonstrated the feasibility of major pulmonary resection via a subcostal approach using an SP robotic system. This technique marked a significant breakthrough by bypassing the intercostal space entirely, thereby eliminating intercostal nerve compression and potentially redefining postoperative pain management in thoracic surgery [2, 3].

In this issue of the *World Journal of Surgery*, Kawaguchi et al. presented a compelling institutional pilot study comparing the initial results of subcostal SP robotic lung resection with the established multi‐port da Vinci Xi platform [4]. The authors should be commended for undertaking this comparative analysis of 60 patients (30 SP vs. 30 Xi), in which no significant differences were found in operative time, console time, blood loss, or the number of lymph nodes harvested. Most notably, the SP group had a remarkably low postoperative complication rate of 6.7%, with zero complications classified as Clavien‐Dindo Grade III or higher.

The authors were quite candid in their discussion about the technical nuances of transitioning to the SP platform. Notably, although the SP system provides flexible graspers and reduces instrument clashing, it currently lacks integrated robotic energy devices, staplers and suction. These critical tasks thus fall to the surgical assistant, increasing their manual burden.

Kawaguchi et al. demonstrated a clear learning curve. By the 24th case, operative times consistently remained under four hours, and the two additional ports that were required in early cases to reach the pulmonary apex were no longer needed. This suggests that for surgeons already proficient in multi‐port RATS, such as the da Vinci Xi, the transition to an SP subcostal approach is safe and achievable. As we look toward the future of robotic thoracic surgery, several key considerations must be addressed before the subcostal single‐port approach can be fully integrated into standard practice:

1. Patient‐centric outcomes: Preserving chest wall integrity is a significant advantage of the subcostal approach. Future prospective trials should prioritize patient‐reported outcomes, specifically comparing long‐term chronic pain and quality of life between subcostal SP and conventional multi‐port Xi approaches.
2. Expansion to complex cases: At present, the SP system's niche is largely confined to less complex procedures. However, as the platform evolves to include robotic stapling and more refined energy devices, its application may expand to complex sleeve resections or cases with dense adhesions—indications that currently favor the multi‐port Xi.
3. Institutional synergy: The SP platform is unlikely to replace the Xi in the immediate future. Instead, institutions may find the greatest value in utilizing both: reserving the Xi for locally advanced cases requiring extensive dissection, while leveraging the SP for anatomical resections where the benefits of “chest‐incision‐free” surgery are most pronounced.

In conclusion, Kawaguchi and colleagues have provided a vital proof of concept for the subcostal da Vinci SP approach. By pioneering the subcostal arch entry point, the authors effectively bypass the intercostal spaces and the associated limitations of the rib cage. Although larger, multicenter trials are necessary to validate these initial findings, this study represents a significant advancement in thoracic surgery. The subcostal single‐port approach is more than a technical novelty: It is a promising evolution toward minimizing surgical trauma and achieving a truly nonintercostal thoracic procedure.

## Author Contributions

**Shah‐Hwa Chou:** conceptualization, formal analysis, writing – original draft, supervision. **Yu‐Wei Liu:** data curation, formal analysis, writing – original draft, writing – review and editing, supervision, funding acquisition.

## Funding

This research was funded by grants from Kaohsiung Medical University Hospital in Taiwan (Grant KMUH114‐4M30).

## Conflicts of Interest

The authors declare no conflicts of interest.

## Data Availability

The data that support the findings of this study are available from the corresponding author upon reasonable request.
